# Therapeutic Antibodies to KIR3DL2 and Other Target Antigens on Cutaneous T-Cell Lymphomas

**DOI:** 10.3389/fimmu.2017.01010

**Published:** 2017-08-30

**Authors:** Christian Schmitt, Anne Marie-Cardine, Armand Bensussan

**Affiliations:** ^1^INSERM U976, Hôpital Saint-Louis, Paris, France; ^2^Paris Diderot University, Sorbonne Paris Cité, Paris, France

**Keywords:** KIR3DL2, Sézary syndrome, mycosis fungoides, cutaneous T-cell lymphomas, tumor marker, monoclonal antibody

## Abstract

KIR3DL2 is a member of the killer cell immunoglobulin-like receptor (KIR) family that was initially identified at the surface of natural killer (NK) cells. KIR3DL2, also known as CD158k, is expressed as a disulfide-linked homodimer. Each chain is composed of three immunoglobulin-like domains and a long cytoplasmic tail containing two immunoreceptor tyrosine-based inhibitory motifs. Beside its expression on NK cells, it is also found on rare circulating T lymphocytes, mainly CD8^+^. Although the KIR gene number varies between haplotype, KIR3DL2 is a framework gene present in all individuals. Together with the presence of genomic regulatory sequences unique to KIR3DL2, this suggests some particular functions for the derived protein in comparison with other KIR family members. Several ligands have been identified for KIR3DL2. As for other KIRs, binding to HLA class I molecules is essential for NK development by promoting phenomena such as licensing and driving NK cell maturation. For KIR3DL2, this includes binding to HLA-A3 and -A11 and to the free heavy chain form of HLA-B27. In addition, KIR3DL2 binds to CpG oligonucleotides (ODN) and ensures their transport to endosomal toll-like receptor 9 that promotes cell activation. These characteristics have implicated KIR3DL2 in several pathologies: ankylosing spondylitis and cutaneous T-cell lymphomas such as Sézary syndrome, CD30^+^ cutaneous lymphoma, and transformed mycosis fungoides. Consequently, a new generation of humanized monoclonal antibodies (mAbs) directed against KIR3DL2 has been helpful in the diagnosis, follow-up, and treatment of these diseases. In addition, preliminary clinical studies of a novel targeted immunotherapy for cutaneous T-cell lymphomas using the anti-KIR3DL2 mAb IPH4102 are now underway. In this review, we discuss the various aspects of KIR3DL2 on the functions of CD4^+^ T cells and how targeting this receptor helps to develop innovative therapeutic strategies.

## Introduction

Introduction of monoclonal antibodies (mAbs) has been a very successful breakthrough for the diagnosis and treatment of a number of tumors. First used for immunophenotyping to better identify and characterize the tumor cell pool, they became useful in the quantification of residual malignant cells during patient follow-up and in the evaluation of chemotherapeutic protocols. mAbs have been highly valuable in identifying therapeutic targets and initiated development of the use of specially designed mAb in cancer treatment. Humanized mAb alone, leaving aside their applications as drug delivery systems, is most widely used for tumor-targeting immunotherapies. Rituximab (anti-CD20 mAb) was the first mAb approved for cancer therapy. It has significantly improved patient survival in several B-cell malignancies such as diffuse large-cell lymphomas with response rate of 60–80% (1, 2). Many efforts have been devoted to understanding the mechanisms of action of anti-CD20 antibody tumor depletion (2–4). Beside complement activation, FcR immune effectors [phagocytes and natural killer (NK) cells] play an essential role in the *in vivo* clearance of mAb-coated tumor cells (5–8). Another way of killing tumor cells by mAb is by F(ab′)_2_-dependent targeting of cell surface signaling receptors associated with apoptosis induction (9–12). In many cases, the therapeutic efficacy of a mAb relies on both Fc- and F(ab′)_2_-dependent mechanisms. However, because of the lack of efficient therapeutic targets and the resistance to chemotherapy, too many cancers are still resistant to treatment, particularly at advanced stages. In this review, we focus on a class of such tumors, the cutaneous T-cell lymphomas (CTCL), which require improved identification of tumor markers and more efficient treatment. The rapidly growing numbers of clinically approved tumor-targeting mAb enlarge the spectrum of potential treatments for these cases.

## Cutaneous T-Cell Lymphomas

Cutaneous T-cell lymphomas represent a group of rare and heterogeneous extranodal non-Hodgkin’s lymphomas characterized by skin infiltration of malignant monoclonal T lymphocytes (13). Sézary syndrome (SS) and mycosis fungoides (MF) are the most common forms of CTCL, both being very difficult to treat at advanced stages. Their diagnosis is based on clinical, histopathological, molecular biological, and immunopathological features (14). However, the lack of unambiguous immunophenotypic or molecular biomarkers makes the differential diagnosis of CTCL with erythrodermic inflammatory dermatoses challenging (15). MF, accounting for around 65% of CTCL cases, usually presents with an indolent clinical course restricted to the skin, passing from macule and patch stage to infiltrated plaque stage. However, tumor-stage disease and cell transformation are associated with much poorer prognosis. SS is an aggressive leukemic variant of CTCL clinically defined by the classical triad of erythroderma, lymphadenopathy, and peripheral blood involvement. Detection of an identical malignant T-cell clone in the skin and the blood, based on T-cell receptor gene rearrangements, is a critical element for the diagnosis of SS. Staging for CTCL based on the TNM (tumor–node–metastasis) system has been extremely useful and remains the standard for the classification of MF/SS patients. Although progress has been made in the treatment of transformed MF and SS, there is still no cure for these diseases. Intensive chemotherapies are mostly inappropriate for CTCL due to the high risk of infection in patients with a compromised skin barrier (14).

As mentioned earlier, early diagnosis of SS can be challenging and evaluation of the tumor burden is difficult. A number of studies have attempted to identify characteristic immunophenotypic changes and molecular biomarkers in Sézary cells that could be useful as additional diagnostic criteria (16–20). Using flow cytometry, the loss of cell surface markers such as CD7, CD26, and/or CD27 on CD4^+^ T cells is helpful to estimate the tumor mass and to orient the choice of therapy. However, the specificity and sensitivity of these tests to identify the malignant clone are to be considered with caution. Markers mostly expressed on NK cells, such as CD158k (KIR3DL2) and CD335 (NKp46), can be expressed on erythrodermic MF/SS T cells and can be considered as more reliable markers for the malignant clone detection (21–24). Despite the possible induction of partial or complete remission, the median survival of SS is 1–5 years, illustrating the need for novel targeted therapies. Promising targets include the C–C chemokine receptor type 4 (CCR4), CD30, programmed-death 1, and KIR3DL2 to which therapeutic mAb has been designed and are currently in the clinical phase of study.

## KIR3DL2 in Biology

### A Biological Marker

In humans, the main NK cell receptors for major histocompatibility class I (MHC-I) molecules are the killer cell immunoglobulin-like receptors (KIRs) or CD158*x*. They have been named according to their biochemical structure, having either two (KIR2D) or three (KIR3D) extracellular Ig-like domains and either a long cytoplasmic tail (KIR-L) containing immunereceptor tyrosine-based inhibitory motifs (ITIM) or a short cytoplasmic tail (KIR-S) that associates with signaling molecules to transduce an activating signal (25). Interestingly, this important function of MHC-I recognition is shared in non-primate species by structurally different molecules, the lectin-like receptors (Ly49*x*). At the genomic level, *KIRs* are encoded in the leukocyte receptor cluster on chromosome 19q13.4, while the *Ly49* genes in rodents are encoded in the NK complex on chromosome 6. Human haplotypes encoding *KIRs* have major differences in gene content and allelic polymorphism, with up to 14 genes and 3 framework genes, namely KIR2DL4, KIR3DL2, and KIR3DL3 (26, 27). Given that MHC-I and KIRs are encoded on different chromosomes, this results in a fascinating complexity of cognate KIRs/HLA class I genotypes.

Unlike T or B lymphocytes, NK cells do not generate their recognition repertoire through receptor gene rearrangements. Instead they use germline-encoded activating and inhibiting receptors, the resulting signals deciding the fate of the cellular response. Expression of a limited set of activating and inhibiting receptors in any given NK lymphocyte ensures the generation of a remarkable degree of cell diversity (28). Inhibiting KIR receptors also play an important role in the development of functional NK cells. The strength of the interaction between the inhibitory KIR and the MHC-I determines the threshold of activation of a given NK cell, a process known as NK cell education (29, 30).

Killer cell immunoglobulin-like receptors are also important in reproduction through the role of uterine NK cells in the process of decidualization (31). During this arterial remodeling process, appropriate KIR/HLA-C interactions between uterine NK cells and extra-villous trophoblasts are necessary to ensure reproductive success (32). For exemple, a strong maternal inhibitory KIR repertoire associated with a fetal high affinity HLA ligand was found to be detrimental to healthy placentation (33).

In the peripheral blood of healthy humans, KIR3DL2 is not only expressed by about 20% of NK cells but also expressed by a small proportion of CD4^+^ (5%) and CD8^+^ (9%) T lymphocytes. An enriched KIR3DL2 expression on memory CD45RO^+^CD28^−^CCR7^−^CD62L^−^ T cells has also been reported (34). Similar to NK cells, KIR3DL2 is an inhibitory co-receptor on T lymphocytes. KIR3DL2 is unique among the KIR family in being expressed as a disulfide-linked homodimer (p140) (35). This characteristic may be important in terms of ligand-binding capacity.

### Ligands

The large number of protein-encoding polymorphic variants of KIR3DL2 hinders definition of the complete list of ligands for this receptor, although the true contribution of allelic variations to ligand recognition is unknown. It was originally shown to bind specifically to HLA-A3 and -A11 (35, 36). It seems that association of the RLRAEAQVK EBV-peptide to HLA-A3 and -A11 is critical for binding to KIR3DL2 (37). The requirement for specific peptide association to the MHC complex is unclear as KIR receptors are more generally considered as MHC-I expression sensors on target cells. This peptide selectivity may confer NK cells with an additional recognition mechanism (38). KIR3DL2 also binds to the free heavy chain dimers of HLA-B27 (39). In addition to the classical ß2m-associated heavy chain, HLA-B27 is expressed at the cell surface as dimers of free heavy chains due to the presence of a reactive cysteine at position 67. In contrast to the binding to HLA-A3 and -A11, KIR3DL2 recognition of HLA-B27-free heavy chains is independent of the bound peptide sequence (40).

KIR3DL2 also binds to CpG-oligodeoxynucleotides (CpG-ODN), and this ligation induces KIR3DL2 down-modulation from the cell surface and translocation to the endosome to deliver the CpG-ODN to the toll-like receptor 9 (TLR9) (41). CpG-ODNs belong to a class of ligands called pathogen-associated molecular patterns (PAMPs) recognized by TLRs. As mentioned earlier, certain TLRs are localized in the endoplasmic reticulum and endosomes where, upon ligation by their respective ligands, they can initiate signal transduction, promote cytokine release, and increase NK cell cytotoxicity (42–45). Recognition of CpG-ODNs or other PAMPs is also observed for KIR3D or KIR2D receptors encompassing a D0 Ig-like domain. This illustrates that KIRs not only function as HLA class I receptors but can also serve as receptors to mediate antimicrobial responses.

### Role in Immune Response

KIR3DL2 belongs to the inhibitory receptor family like the other KIR-L and is characterized by the presence of ITIM/ITSM-like sequences in its cytoplasmic tail. It is indeed capable, upon ligation at the surface of NK cells, to inhibit IFNγ production and cytotoxic function. On T lymphocytes, KIR3DL2 ligation has no effect alone. It is a co-receptor that contributes to the response initiated *via* the TCR. In particular, KIR3DL2 ligation on activated T cells results in an antiapoptotic effect and the production of IL-17 (46). KIR3DL2 ligation by HLA-B27 also promotes the survival of NK cells and inhibits their production of IFN-γ (34). Of note, KIR3DL2 expression is upregulated upon activation of NK and T cells. KIR3DL2 expressing T cells may therefore be enriched in Th17 cells, the T-cell subset producing IL-17, suggesting that it may have a role in the differentiation of this T-cell subset. Interestingly, while IL-17 can have anti-tumor effects as a pro-inflammatory cytokine, it has also been identified as exerting a promoting role in carcinogenesis, tumor metastasis, and resistance to chemotherapy of diverse types of cancers (47).

## KIR3DL2 in Pathology

### KIR3DL2 and Ankylosing Spondylitis (AS)

The human leukocyte antigen HLA-B27 is strongly associated with the development of AS, with 94% of patients expressing HLA-B27, compared to 9.4% of healthy individuals (48). As mentioned earlier, KIR3DL2 recognizes HLA-B27 as a dimer of free B27 heavy chains. Proportions of KIR3DL2^+^ CD4^+^ T cells producing IL-17 are increased in the peripheral blood and synovial fluid of patients with AS (34). *In vitro*, KIR3DL2^+^ CD4 T cells stimulated with B27 heavy chain dimers or IL-23 and IL-1 produce more IL-17 when isolated from AS patients than from healthy individuals (46). A role for Th17 cells in the pathogenesis of AS has been suggested by the strong genetic linkage with IL-23R polymorphism (49). In AS, KIR3DL2^+^CD4^+^ T cells accounted for 60% of all IL-23R-expressing CD4^+^ T cells. These data suggest that the B27 interaction with KIR3DL2 could play a central role in AS and other HLA-B27-linked autoimmune diseases.

### KIR3DL2 in CTCL

#### A Diagnostic Tool

As mentioned earlier, diagnosis and evaluation of the tumor mass in CTCL can be challenging. Histological examination of blood smears to determine the tumor mass, with Sézary cells defined by a cerebriform nuclear morphology, is widely used and valuable, while flow cytometry analysis of T-cell blood subsets provides a more objective and reproducible means to quantify and track circulating lymphocyte involvement in patients with MF/SS. For example, a CD4:CD8 ratio higher than 10 is observed in about 80% of patients with SS, whereas loss of CD7 (CD4^+^CD7^−^ ≥30%) or CD26 (CD4^+^CD26^−^ ≥40%) is found in about half of the SS patients (50–52). However, loss of CD7 or CD26 among CD4^+^ T cells can also be found in benign inflammatory erythroderma or rare healthy subjects. Even T-cell clonality can be detected in 34% of cases with benign inflammatory erythroderma (53). This illustrates the need for other specific markers for CTCL. Among the proposed potential markers, several belong to the NK cell lineage, raising the provocative question of a NK-cell reprogramming mechanism occurring in the transformation of some CTCL (54). Indeed, an abnormal expression of several NK receptors has been observed at the surface of SS cells. These include CD85j/Ig-like transcript 2 (ILT2)-receptor (55), the natural cytotoxicity receptor (NCR) NKp46/NCR1 (22), and KIR3DL2 (56).

Ig-like transcript 2 is an inhibitory receptor, analogous to KIRs, specific for the α3-domain epitope shared by some MHC-I molecules and the UL18 antigen encoded by human cytomegalovirus (57). Although it is expressed on NK and some memory CD8^+^ T cells, ILT2 is absent from the surface of resting normal CD4^+^ T lymphocytes, allowing ILT2 expression to effectively identify circulating Sézary cells in SS patients (55).

NKp46, with NKp30 and NKp44, constitutes the NK cell NCRs family. It was shown that umbilical cord blood CD8^+^ T cells long-term cultured with IL-15 could express NCR (58). In this line, NCRs are also observed on the surface of intraepithelial T lymphocytes in celiac disease where they can drive TCR-independent cytotoxicity and cytokine production (59). It was still a surprise to find NKp46 expression not on cytotoxic effector T cells but on non-CTL malignant CD4^+^ T lymphocytes in SS patients (22). Expression of NKp46 is not observed on normal circulating CD4^+^ T cells, a clear indication that such ectopic expression is a consequence of malignant transformation. Indeed, in the circulating T cells from SS patients, expression of NKp46 is restricted to the clonal Vß CD4^+^ population identifying the tumor cells. NKp46 expression also correlated with KIR3DL2 expression and reflected the clinical course of the disease with a lower expression in remission or post-treatment periods. Of note, NKp46 and KIR3DL2 are frequently co-expressed in transformed MF (23).

The Q66 mAb was the first to specifically recognize the p170 KIR now called CD158k or KIR3DL2 (35). Although SS cells are difficult to culture *in vitro*, IL-7 proved to be useful to generate some CTCL cell lines (60–62). Expression of KIR3DL2 detected by the Q66 mAb was initially demonstrated in the former cell lines and further confirmed on the corresponding patient’s primary circulating cells and extended to seven other SS patients and to the skin of two patients with advanced MF (56). Expression of KIR3DL2 on SS cells is polymorphic but is not associated with a particular allele (63). It is increasingly clear that KIR3DL2, alone or associated with other markers, could efficiently delineate circulating Sézary cells and reduce diagnostic difficulties (21, 64, 65). Because KIR3DL2 is poorly expressed by normal T lymphocytes or reactive lymphocytes from benign erythrodermic inflammatory diseases, it is a highly specific marker for Sézary cells. For example, KIR3DL2 transcripts were found significantly overexpressed in skin biopsies from patients with SS compared to benign erythrodermic dermatoses (66). KIR3DL2 has progressed to become the best marker of SS after expression was reported in 30 of 34 (82%), 32 of 33 (97%), and 11 of 17 (65%) patients with SS in studies from three different groups (51, 64, 67). Availability of new IgG anti-KIR3DL2 mAb with higher affinity and avidity than the first IgM Q66 will increase the possibility of detecting SS cells with low CD158k/KIR3DL2 expression. KIR3DL2 mAb identifies the tumor cells in most CTCL patients analyzed and, therefore, represents a valid tool to dynamically evaluate the evolution of the tumor pool during disease evolution as well as the response to treatment (68).

#### A Prognostic Tool

During the course of the disease or after treatment, it is crucial to assess whether an increase in the CD4 population results from an expansion of the tumor cell population or of reactive T lymphocytes. In addition, the tumor burden in blood had a prognostic value in patients with eythrodermic CTCL (69). It was shown that measure of the absolute CD3^+^CD158k^+^ circulating cells closely correlated with morphologically identified (with cerebriform-like nuclei) Sézary cell numbers in patients with SS under systemic therapy (21). When comparing KIR3DL2^+^ and KIR3DL2^−^ populations of CD4^+^ T cells, a decrease in KIR3DL2 expressing T cells is associated with the response to therapy when increased KIR3DL2^−^ CD4^+^ T cells are observed, highlighting the usefulness of this marker for the follow-up of SS patients (17). A recent study reported that 87% of a group of SS patients (*n* = 64) expressed KIR3DL2 (range: 7–98% of tumor T cells) at diagnosis. Analyzing the follow-up of these patients indicates that the presence of more that 85% of KIR3DL2^+^ cells among CD3^+^ T cells is the main prognostic factor at diagnosis for SS (68). This study also demonstrates that circulating CD3^+^CD4^+^KIR3DL2^+^ T-cell counts can be used to monitor treatment efficacy and relapse in SS patients. KIR3DL2 detection permits to estimate whether the ongoing treatment specifically targeted the malignant T-cell clone and, if so, to visualize the pool of residual tumor cells. KIR3DL2 therefore represents an early predictive marker of relapse or progression.

#### A Therapeutic Target

Based on the above data, it is clear that KIR3DL2 can be a therapeutic target for CTCL. However the remaining questions are: why do cutaneous malignant T cells express this marker and what is its function on these cells? As mentioned earlier, KIR3DL2 expression on CTCL cells may be the result of some kind of genetic remodeling that induces NK marker ectopic expression. In healthy individuals, KIRs were also expressed by a small proportion of cytotoxic CD8^+^ T cells (70). Unlike NK cells, where inhibitory KIRs play a role in education, KIRs on T cells are acquired at the memory stage, and the proportion of KIR^+^CD8^+^ T lymphocytes increases with age (70). The engagement of MHC class I inhibitory receptors has been shown to contribute to the down-regulation of T cell effector function in KIR^+^CD8^+^ T and to the negative control of activation-induced cell death (AICD) (71). Expression of KIRs (or ILT2) can proceed from a regulatory mechanism that would raise the activation threshold in cytotoxic T cells, thereby providing a safety mechanism to control these potentially harmful cells (72). Therefore, KIR3DL2 expression on Sézary cells may reflect the ability of neoplastic cells to better avoid antigen receptor-mediated cell death in an inflammatory environment of persistent antigenic stimulation of cutaneous T cells. Indeed, it has been established that accumulation of Sézary cells is not a result of increased proliferation but rather reflected a resistance to apoptosis and particularly to AICD (73). Thus, although KIR3DL2 has no independent receptor function, co-ligation of CD3 and KIR3DL2 induces a strong inhibition of the proliferation and apoptosis on Sézary cells when non-KIR3DL2 cells proliferated and reached apoptosis normally (74). Thus, expression of KIR3DL2 by malignant cutaneous T cells may be a key element to protect these cells from AICD in a highly stimulating environment, but what can trigger KIR3DL2 signaling in Sézary cells? There is no particular association of SS with the HLA-A3, -A11, or -B27 haplotype but, as mentioned earlier, CpG-ODN has been reported to bind KIR3DL2 on NK cells leading to NK cell activation (41). When tested on Sézary cells, KIR3DL2 engagement by CpG-ODN promotes the internalization of the receptor and the generation of apoptotic signals in the malignant CD4^+^ cells (75). In phase I/II trials on CTCL patients, subcutaneous injection of class-B CpG-ODN has led to a clinical response rate of 32–36% and in the absence of major cytotoxic side effects (76, 77). These data suggest that, in addition to promoting the generation of an anti-tumor immune response, CpG-ODN might initiate a direct effect on Sézary cells through binding to KIR3DL2.

KIR3DL2 expression is not restricted to SS and MF tumor cells. Recently it has been shown that in primary anaplastic large-cell lymphoma, an aggressive CD30^+^ CTCL, tumor cells also express KIR3DL2 and can be the target of a potent anti-tumor activity *in vitro*, re-enforcing this marker as a therapeutic target for these patients (78). The restricted expression of KIR3DL2 in normal immune cells, and the possibility to selectively target the tumor cells in CTCL, led to the development of a humanized mAb for an effective treatment strategy. IPH4102, a humanized IgG1, is the selected anti-KIR3DL2 mAb candidate with potent depleting activity against primary Sézary patient cells (79, 80). In preclinical studies, *in vitro* assays using the Sézary cell line HuT 78 demonstrated that IPH4102 modes of action include antibody-dependent cell cytotoxicity (ADCC) involving NK cells, and antibody-dependent cell phagocytosis due to macrophage activation, but no killing *via* direct complement activation on target cells (79). Effective anti-tumor killing was also achieved *ex vivo* in co-cultures of patient’s Sézary cells and autologous NK cells as effectors. Even with an effector/target ratio lower than 1:1, malignant cells from all patients tested were efficiently killed while NK effector survival was not compromised by the action of IPH4102, unlike the Alentuzumab control, illustrating the selectivity of action of the anti-KIR3DL2 mAb (79). *In vivo* efficiency of the anti-tumor activity of IPH4102 was tested in SCID mice engrafted with KIR3DL2-transfected Raji cells. In this model, engrafted mice treated twice weekly with an isotype control mAb had a median survival of 17 days. On the other hand, mice treated with as little as 30 µg of IPH4102 had a median survival of 39 days, and 2 of the 30 mice remained alive at the end of the experiment (79). IPH4102 has also demonstrated a favorable preclinical safety profile in regulatory pharmaco-toxicology experiments in non-human primates (80).

IPH4102 is currently being investigated in the first-in-human multicenter phase I study (NCT02593045) evaluating repeated administrations at escalating doses of single-agent IPH4102 in relapsed/refractory CTCL. A recent report of this ongoing study that includes 22 CTCL patients, including 19 SS patients, indicated that IPH4102 is very well tolerated in these patients (who have had extensive treatment) with no reported treatment-related death (81). The majority of adverse effects are low grade and typical for CTCL. Upon escalation and up to now, the best global overall response rate is 47% in SS patients, reaching 58% responses in the circulation. In addition, two complete responses were observed in skin and five complete responses were observed in blood. These preliminary data are encouraging to promote IPH4102 as a new targeted treatment in patients with advanced CTCL.

## Other mAb in CTCL Treatment

### Alentuzumab (Campath; Anti-CD52 mAb)

Alemtuzumab is a humanized IgG1 kappa mAb specific for CD52, an antigen expressed by most T and B lymphocytes. The usual protocol of administration of 30 mg doses three times per week has led to a good outcome in SS, but much less convincing results in patients with MF. However it was associated with severe leukopenia, immune depletion and opportunistic infections that may require treatment discontinuation (82, 83). To minimize immune suppression and infections due to its wide expression in the immune system, protocols have been proposed with injection of 10 mg only if SS cells become higher than 1,000/mm^3^ (84). Although side effects are considerably reduced, such protocols are usually not curative on a long-term perspective.

### Brentuximab Vedotin (Anti-CD30 mAb)

Brentuximab vedotin (SGN-35) is a chimeric anti-CD30 mAb conjugated to monomethyl auristatin E, a cytotoxic anti-tubulin agent. Thirty-two MF or SS patients at stage IB-IV were included in a phase II prospective study (85). The overall response rate was 70% with responses at all stages. However, the expression of CD30 by immunochemistry was very variable and patients with expression lower than 5% experienced decreased probability of response. Another prospective phase II study, targeting 48 patients with CD30^+^ CTCL and including 28 CD30^+^ MF, reported an overall response rate of 71% with a complete response in 35% of cases (86). In these studies, the most frequent adverse event is peripheral neuropathy, occurring in 66% of the patients and showing resolution during a 2-year time course.

### Mogamulizumab (Anti-CCR4 mAb)

Mogamulizumab is a humanized anti-CCR4 monoclonal antibody with a defucosylated Fc region leading to increased antibody-dependent cellular cytotoxicity (87). CCR4 is expressed on Tregs and T helper cells and plays an important role in skin homing. In a phase I/2II study, mogamulizumab induced an overall response rate of 47.1% in SS patients and 28.6% in MF patients (88). In a multicenter Japanese phase II study involving 37 patients with relapsed CCR4-positive tumors, mogamulizumab treatment induced 35% of objective response, including 5 patients (14%) experiencing complete response (89). The most common adverse effect of this treatment is lymphocytopenia (81%), and cases of severe Steven–Johnson–Lyell syndrome due to the induced immune deficiency of regulatory T cells have been reported (90, 91). An international phase III trial of mogamulizumab versus vorinostat is ongoing in previously treated CTCL patients.

## Conclusion

There is no doubt that the development of targeted systemic biological therapies will benefit the management of CTCL. Several ongoing trails with therapeutic mAbs, including brentuximab vedotin, mogamulizumab, and IPH4102, show interesting results in these patients with limited toxicity. KIR3DL2 is expressed irrespectively of disease stage in all subtypes of CTCL, with the highest prevalence in SS and transformed MF, two subsets with high and unmet therapeutic needs. The origin of its expression is unknown, but the presence of chronic bacterial stimulation may be responsible for increased KIR3DL2 expression on cutaneous T cells. The limited expression of KIR3DL2 on normal immune cells, in comparison with the ectopic expression on CTCL tumor cells, allows to selectively and efficiently kill malignant cells. KIR3DL2 can be targeted by both IPH4102 or by one of its ligands, CpG-ODN. The mAb acts *via* an ADCC and phagocytosis, while CpG-ODN induces the internalization of KIR3DL2 receptor upon ligation and cell apoptosis *via* the Toll-like receptor activation pathway. CpG-ODN also efficiently activates key elements of the immune system that participate in the local anti-tumor rejection process. One can hope that the identification of novel targets and development of therapeutic mAbs will prove to be efficient and safe alternatives for the treatment of CTCL.

## Author Contributions

AB and AM-C are senior co-authors. AB, AM-C, and CS wrote the manuscript.

## Conflict of Interest Statement

The authors declare that the research was conducted in the absence of any commercial or financial relationships that could be construed as a potential conflict of interest.
